# Endoscopic non-ablative fractional laser therapy in an orthotopic colon tumour model

**DOI:** 10.1038/s41598-018-19792-2

**Published:** 2018-01-26

**Authors:** Su Woong Yoo, Gyungseok Oh, Abdul Mohaimen Safi, Soonjoo Hwang, Young-Seok Seo, Kyung-Hwa Lee, Young L. Kim, Euiheon Chung

**Affiliations:** 1 Department of Nuclear Medicine, Chonnam National University Hwasun Hospital, Jeollanam-do, Republic of Korea; 2 Department of Biomedical Science and Engineering, Institute of Integrated Technology (IIT), Gwangju Institute of Science and Technology (GIST), Gwangju, Republic of Korea; 3 School of Mechanical Engineering, Gwangju Institute of Science and Technology (GIST), Gwangju, Republic of Korea; 4 R & D center, WONTECH Co., Ltd, Daejeon, Republic of Korea; 5 Department of Pathology, Chonnam National University Hwasun Hospital and Medical School, Jeollanam-do, Republic of Korea; 6 Weldon School of Biomedical Engineering, Purdue University, West Lafayette, IN USA

## Abstract

Colorectal cancer is one of the leading causes of cancer-related deaths. Although several therapeutic management strategies are available at the early colon cancer stages, such as endoscopic mucosal or submucosal dissection, associated complications often include bleeding or bowel perforations. As an alternative approach, we investigated endoscopic non-ablative fractional laser (eNAFL) irradiation as a minimally invasive therapeutic modality for the treatment of early-stage colorectal cancer. By implanting SL4-DsRed colon cancer cells into the colons of the C57BL/6 mice, we developed an orthotopic colon tumour mouse model and demonstrated the early-stage tumour growth delay following the eNAFL irradiation. Additionally, we evaluated the temperature changes in the eNAFL-irradiated area using numerical simulations, and induced inflammation using histological analysis. Our results indicate a minimal thermal damage confined to the irradiated spot, sparing the adjacent tissue and alteration in the tumour microenvironment. eNAFL irradiation may be clinically useful as a minimally invasive therapeutic intervention at the early stage of tumourigenesis. In future, an optimal eNAFL therapeutic dose should be determined, in order to increase the efficacy of this approach.

## Introduction

Colorectal cancer is the second most frequent cause of cancer-related deaths in the US^1^, and colonoscopy is firmly established as the mainstay of cancer prevention. Early-stage colon cancer is defined as cancer that is confined to the mucosa or submucosa and does not invade muscularis propria^2^. Currently, several therapeutic options for the early-stage colorectal cancer therapy using the endoscopy are available, such as snare polypectomy^3^, endoscopic mucosal resection^4^, and endoscopic submucosal dissection^5^. However, these treatment strategies are associated with complications such as bleeding or bowel perforations^6–8^. Additionally, despite the endoscopic tumour resection, residual tumour can be found, and approximately 13.1% of polyp recurrence rate was observed after endoscopic mucosal resection^9^. Other therapeutic options include radiation therapy, chemotherapy, immunotherapy, and targeted therapy, and a combination of these treatments may lead to the improvement in the progression-free or overall survival rates^10–12^. However, these approaches are primarily used for the treatment of the advance-stage colon cancer rather than the early-stage colon cancer^13^. Therefore, it would be beneficial for developing a treatment method that could easily be integrated with conventional endoscopic systems to suppress early tumour growth in a minimally invasive manner.

We investigated non-ablative fractional laser (NAFL) as an alternative therapeutic modality. NAFL delivers infrared-wavelength non-ablative laser light to the tissue with pixelated pattern, and this treatment selectively induces wound response in the dermal tissue, while minimizing the damage to the epidermis, in contrast with the ablative laser treatments, which remove the epidermis of the irradiated area^14^. Currently, both ablative and NAFLs are widely used as a medical device. By replacing geriatric tissue with the newly-developed one, the texture and colour of the skin were shown to be significantly improved after irradiation^15^. Although the non-ablative laser treatment is less invasive, it was shown to have a limited therapeutic efficacy^16^, and fractional laser was developed to enhance the therapeutic efficacy, as it delivers high-energy light to the skin in a pixelated pattern, whereas non-fractionated lasers irradiate the entire projected area^17,18^.

Recently, it was reported that fractional lasers can be used for skin cancer prevention, showing that, following repeated irradiations of the mouse skin by the fractional laser, tumour occurrence rate and tumour progression in the skin of mice maintained under ultraviolet light, were significantly lower than those in the non-irradiated skin^19^. These preventive effects may be useful for other epithelial tissues, such as colon and rectum, as well.

We recently reported the therapeutic efficacy of NAFL irradiation in a subcutaneous tumour model^20^. However, the ectopic model (colon cancer cells growing in skin) was shown to have limitations due to the differences in the tumour microenvironment^21,22^. Tumour response to therapy can be different depending on the localisation of the tumour^23^. Genetically engineered mouse models of cancer usually require a long time to develop tumours and the frequency, time, and location of primary tumours are less predictable^22^. Therefore, the use of the orthotopic tumour model with natural tumour environment is beneficial, as it is more predictable.

In this study, we developed an endoscopic NAFL (eNAFL) irradiation system to determine its therapeutic effects on the early-stage tumours. Initially, we developed an orthotopic colon tumour model, matching the original anatomic location of the tumour by implanting SL4-DsRed cancer cells into the colon of C57BL/6 mouse. Following this, we examined the preclinical usefulness of fractional laser as a cancer therapeutic modality using the developed animal model. Additionally, using the numerical simulation and histological analysis, we examined the physiological processes induced by laser irradiation. Additional thermal imaging was performed to evaluate temperature changes in real time. To the best of our knowledge, this is the first study reporting the use of a NAFL as an early cancer therapeutic modality in an endoscopic setting.

## Results

### Development of orthotopic colon tumour model

**Figure 1:**
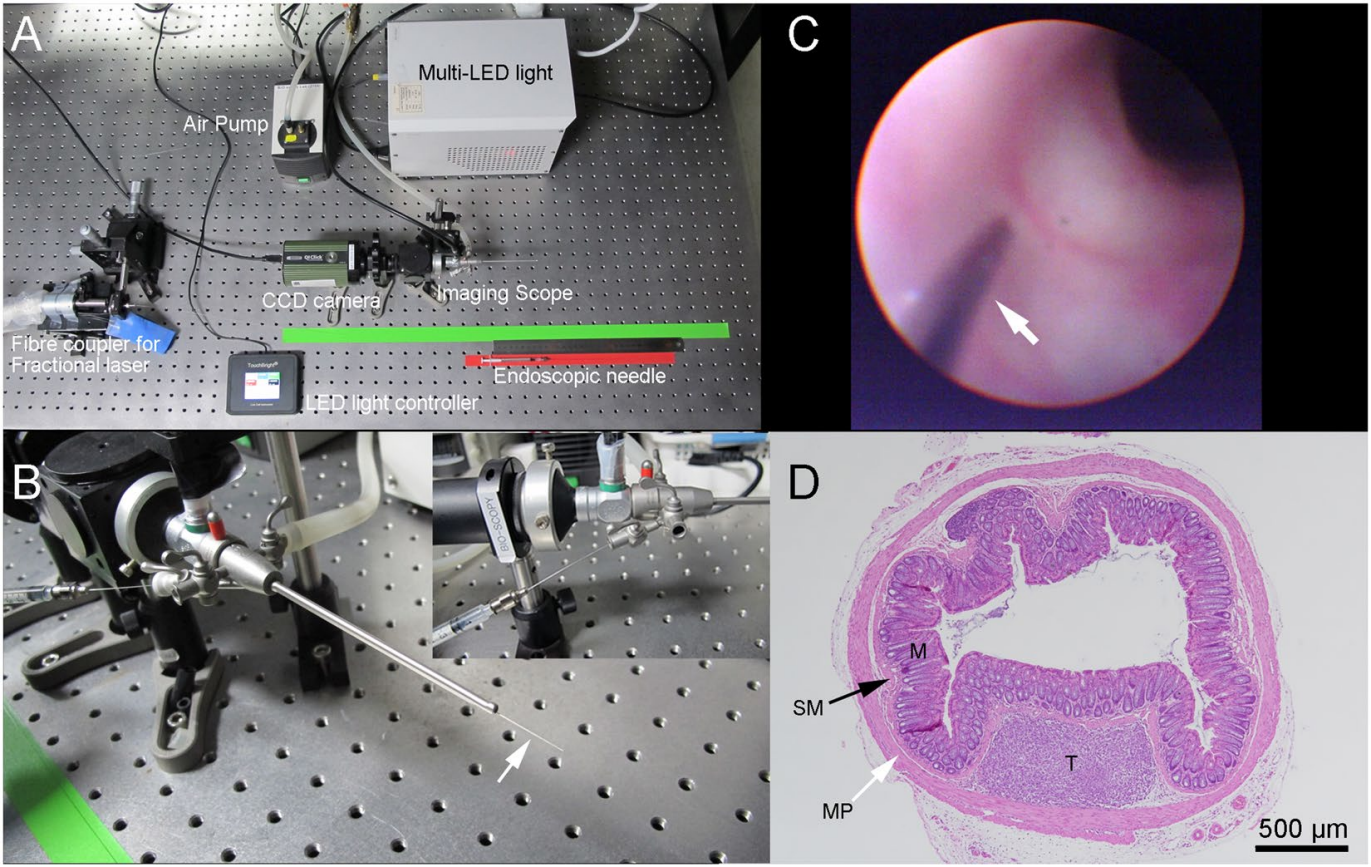
Development of the orthotopic mouse colon tumour model. (**A**) Endoscopic non-ablative fractional laser and endoscopic needle system. (**B**) Eight-inch needle (white arrow) was inserted thorough the instrument channel of endoscopic system. (**C**) Endoscopic implantation of colon cancer cells into the colonic wall using the needle (white arrow) *in vivo*. (**D**) Haematoxylin & eosin staining of the implanted cancer cells. Tumour cells (T) grew in the submucosal (SM) space. Mucosa (M), submucosa (SM, black arrow), and muscularis propria (MP, white arrow) are presented.

In Table 1, an overall summary of three cancer cell injection methods used to make an orthotopic tumour model is presented. The success rates of intra-luminal seeding with laparotomy, intra-colonic wall injection with laparotomy, and endoscopic implantation of colorectal cancer methods were 20.0%, 75.0%, and 92.3%, respectively. Because of the various outcomes, different numbers of follow-up days following the cancer cell implantation were used. General eNAFL system is presented in Fig. 1A. Endoscopic implantation methods (Fig. 1B–D and Supplementary Video S1) led to the highest success rate, resulting in a short follow-up time without the requirement for laparotomy, a major surgical procedure. Therefore, we selected the endoscopic implantation method for the generation of the orthotopic colon tumour model.

**Table 1:** Comparisons between the methods used to make the orthotopic tumour model.

Methods	Success rate (%) (# of successful attempts /total used mice)	Last follow-up (days)	Advantages	Disadvantages
Intra-luminal seeding with laparotomy	20.0 (3/15)	45	Simultaneous introduction of a large number of cancer cells	Laparotomy is necessary; Complicated surgical procedure
Intra-colonic wall injection with laparotomy	75.0 (6/8)	21	Relatively simple procedure	Laparotomy is necessary; High rate of failure of the intracolonic injections
Endoscopic implantation	92.3 (12/13)	10	Minimally invasive; Relatively faster tumour growth	Endoscopic tools are necessary; High rate of failure of the intracolonic injections

### Tumour growth delay induced by the eNAFL irradiation

**Figure 2:**
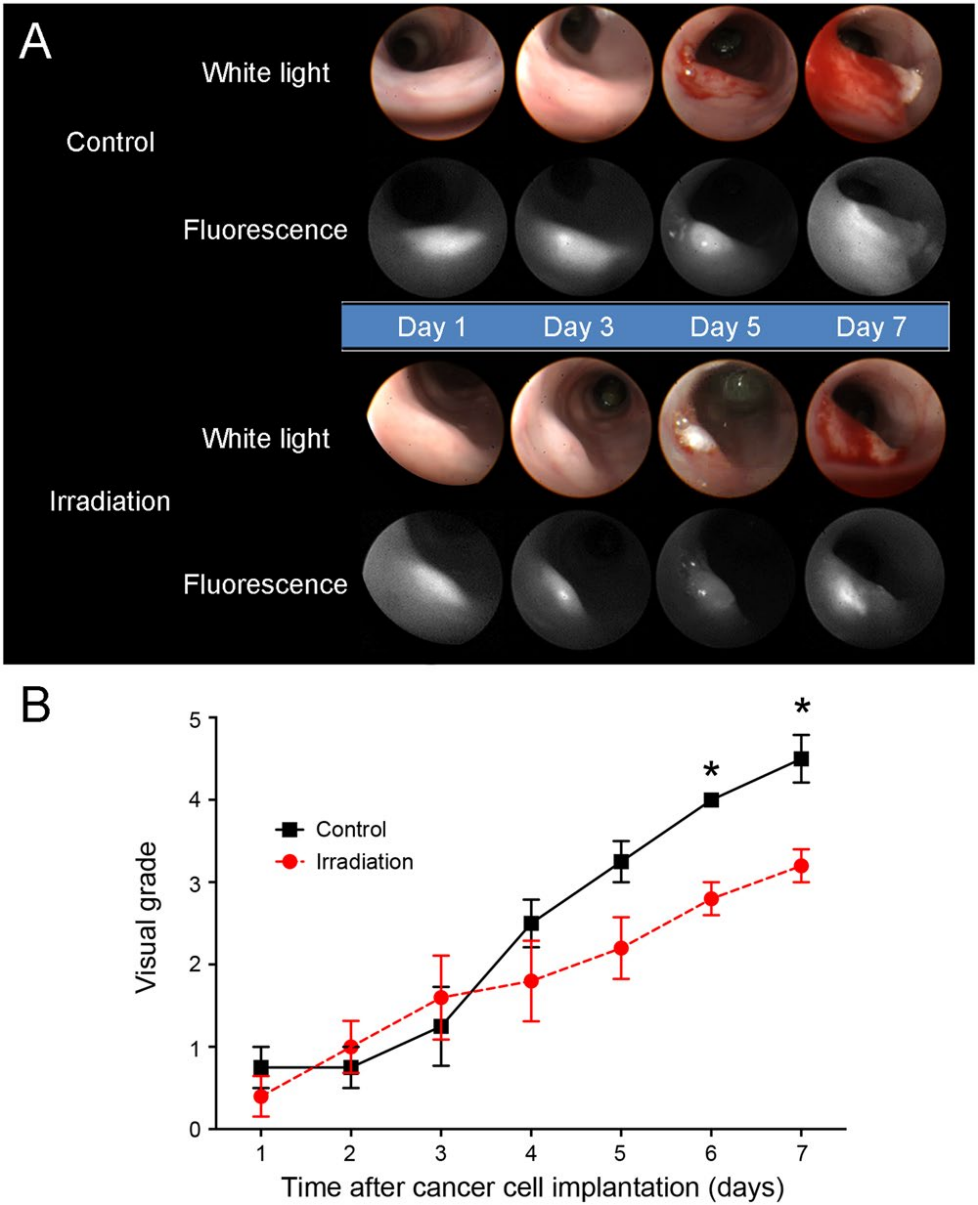
Therapeutic efficacy of the endoscopic non-ablative fractional laser (eNAFL) irradiation. (**A**) Endoscopic images of the orthotopic tumour models after eNAFL irradiation. Representative white light and fluorescence images of the control and eNAFL-irradiated mice are presented. (**B**) Blinded visual grading of the tumours in the orthotopic tumour model mice. eNAFL-irradiated group (n = 5) was shown to have lower average tumour grades than the control group (n = 4). *p < 0.05 (Mann-Whitney U test). All results are presented as mean ± standard deviation (SD).

We performed endoscopic imaging of the colonic walls in the control (n = 4) and eNAFL irradiated group (n = 5) every day for 1 week (Fig. 2A). Both white light and fluorescence imaging were performed to assess the tumour growth. Lower tumour growth rates were observed in the eNAFL-irradiated group than those in the control group according to both imaging modalities. Visual scoring of the tumour growth based on the white light images revealed lower tumour grades in the eNAFL-irradiated group (Fig. 2B). Statistically significant differences between the two groups were observed at day 6 (p = 0.0079) and 7 (p = 0.0317) after cancer cell implantation.

### Temperature distribution following the eNAFL irradiation, determined by using the numerical simulation

**Figure 3:**
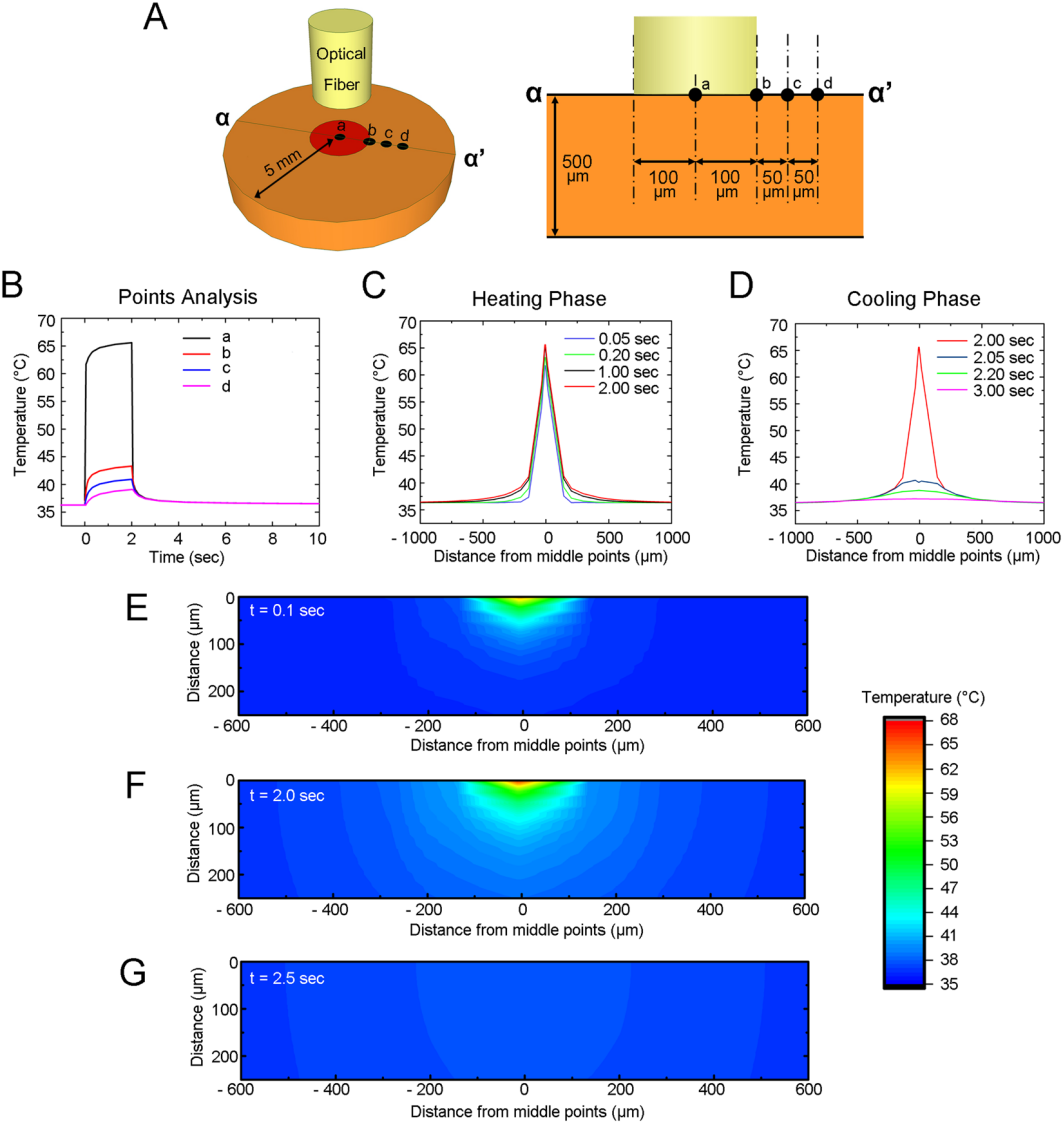
Numerical simulation of the endoscopic non-ablative fractional laser (eNAFL) irradiation of a colon. (**A**) Left: 3D image of the tissue model used for numerical simulation. Right: Cross-cut image of the 3D tissue model with the α-α′ plane. (**B**) Temporal analysis of temperature changes in four representative spots. (**C** and **D**) Spatial temperature distribution during heating (**C**) and cooling (**D**) periods in the tissue model. (**E**–**G**) Temperature distributions at different time points during and after laser irradiation. Cross sections were made through the centre of the tissue model.

To estimate the thermal effect of the eNAFL irradiation, we developed a computational numerical simulation model with a single irradiation spot on a tissue sample (Fig. 3A). In the simulation, a flat surface was assumed since the optical fibre tip was in full contact with the colonic mucosa during the irradiation. Although we irradiated four spots on the tumour surface, the simulation using only one irradiation spot should reflect the actual physical process, as we sequentially irradiated each spot. For detailed thermal analysis, four points (a, b, c, and d) were selected at the distances of 0, 100, 150, and 200 µm, respectively, from the centre of the irradiation spot. The temporal changes in temperature at these four representative points show that the highest temperature increase occurs at the centre of the eNAFL irradiation spot (point a), while the lowest temperature changes were observed in the point d (Fig. 3B). The peak temperature reaches approximately 65.5 °C at t = 2 s, after laser irradiation. Spatial temperature distribution within the incident plane containing the line α-α′ shows a high-temperature peak at the centre of the eNAFL irradiation spot during the heating and the natural cooling phases (Fig. 3C and D). After 2 s, the temperature was shown to decreases rapidly in the adjacent tissue (Fig. 3D, cooling phase). Temperature distribution at different time points (Fig. 3E–G) along cross-cut tissue images showed a minimal thermal propagation to the adjacent tissue, while the highest temperature was confined to the centre of the irradiation spot.

### Histological analysis of the eNAFL-irradiated tissue changes with time

**Figure 4:**
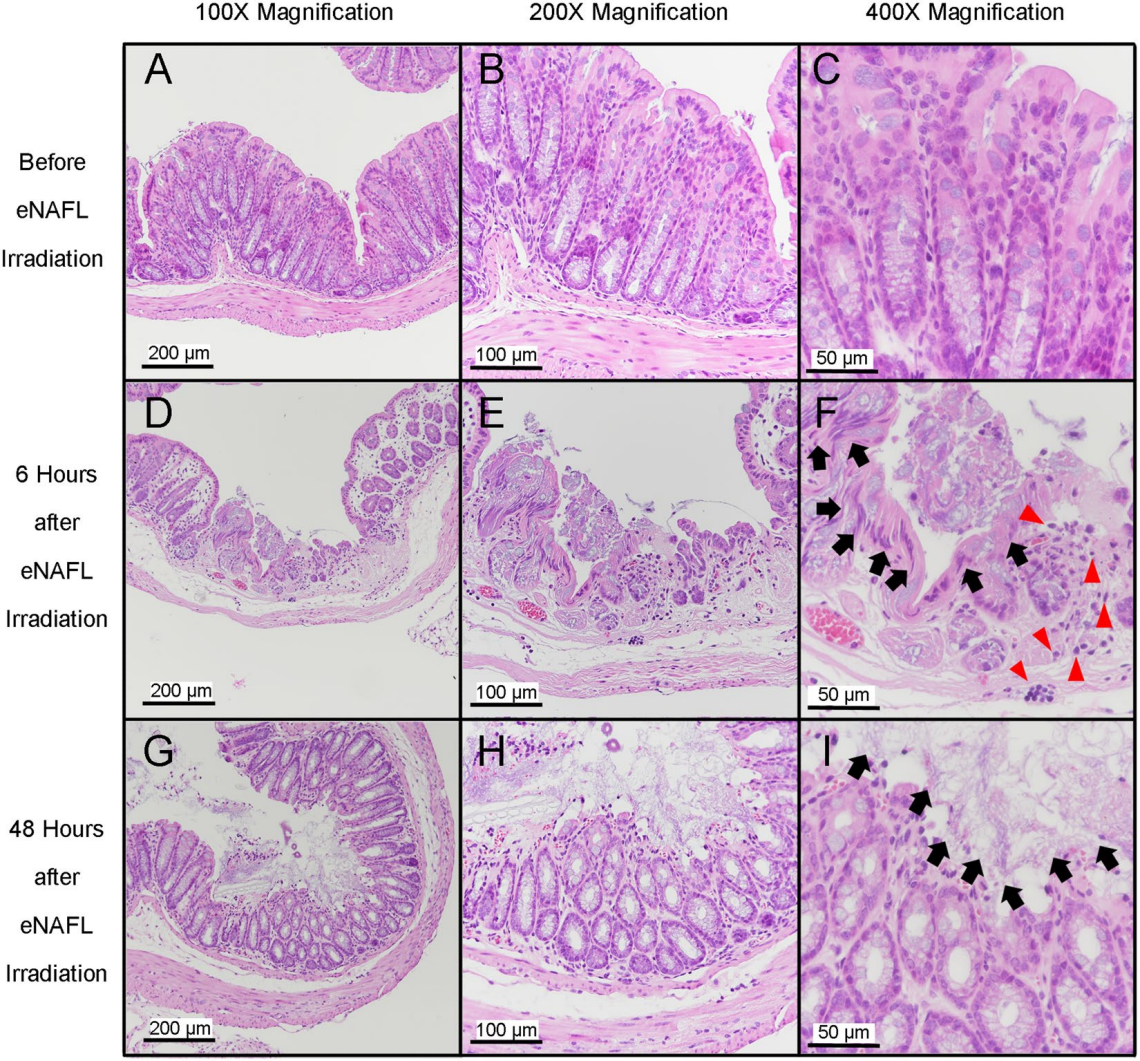
Histological changes induced by the endoscopic non-ablative fractional laser (eNAFL) irradiation in the normal mouse colons. Haematoxylin & eosin (H&E) staining images were obtained with at 100× (**A**,**D**,**G**), 200× (**B**,**E**,**H**), and 400 × (**C**,**F**,**I**) magnification. (**A**–**C**) Tissue images before the eNAFL irradiation, with the normal glandular structure. (**D**–**F**) Tissues at 6 h after eNAFL irradiation. Black arrows, damaged nuclei; red arrowheads, immune cell infiltration. (**G**–**I**) Tissue images at 48 h after eNAFL irradiation. Black arrows, diffusely erosive mucosal layer.

Haematoxylin & Eosin (H&E) staining of the colon specimens from control and tumour-bearing mice was performed. In healthy mice, the colonic structure was shown to be preserved prior to the eNAFL irradiation (Fig. 4A–C). However, considerable mucosal changes were observed 6 h after the irradiation (Fig. 4D–F), together with the elongated nuclei cauterised by thermal damage and necrotic glands (Fig. 4F, black arrows). Many neutrophils were shown to infiltrate into the mucosa and submucosa (Fig. 4F, red arrowheads). After 48 h, the eNAFL-irradiated tissues showed erosive changes in the healing processes, including a mild epithelial detachment and muco-inflammatory exudates (Fig. 4G and H, and black arrows in Fig. 4I), together with the removal of the leukocytes from the mucosa.

**Figure 5:**
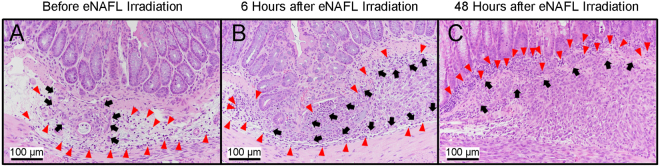
Histological changes in the orthotopic colon tumour mouse tissues after endoscopic non-ablative fractional laser (eNAFL) irradiation. Haematoxylin & eosin (H&E) staining images were obtained at 200× magnification. (**A**) Tumour tissue before the eNAFL irradiation. Black arrows, tumour cells located in the submucosal space; red arrowheads, immune cell infiltrations. (**B**) Colon tissue at 6 h after the eNAFL irradiation. Red arrowheads, many infiltrated immune cells; black arrows, tumour cells. (**C**) Colon tissue, at 48 h after the eNAFL irradiation. Black arrows, tumour cells invading mucosal tissue; red arrowheads, peripherally accumulated immune cells.

In mice with the orthotopic colon tumours, similar histological changes were observed (Fig. 5). Prior to the irradiation, only some neutrophils (Fig. 5A, red arrowheads) were present near the tumour clusters implanted 24 h earlier (Fig. 5A, black arrows). Six hours after the eNAFL irradiation, an increased number of inflammatory cells comprising mononuclear cells and neutrophils (Fig. 5B, red arrowheads) were identified around the tumour nest (Fig. 5B, black arrows). After 48 h, immune cells (Fig. 5C, red arrowheads) were observed at the periphery of the growing tumour mass (Fig. 5C, black arrows).

## Discussion

We developed a novel eNAFL irradiation modality and examined its growth delay effects by using an orthotopic tumour model. To the best of our knowledge, this is the first endoscopic application of the thulium NAFL for the therapy of the gastrointestinal tract cancer. eNAFL irradiation resulted in statistically significant tumour growth delay. Although these results are consistent with those of a previous study that used the subcutaneous skin tumour model^20^, the results of our approach indicate an improved clinical significance against orthotopic tumour growth in an endoscopic setting.

To create a reproducible orthotopic tumour model, we investigated different approaches, and showed that the cancer cell implantation mediated by the intra-colonic wall injection with laparotomy has a tumour-formation success rate comparable with that of the endoscopic implantation method. However, a dexterous procedure is required to inject cells into the submucosal layer of the thin colon, and the proper implantation of cancer cells is essential in this preclinical approach. In this study, the endoscopic implantation method resulted in a higher success rate. As a minimally invasive procedure, the advantage of this method is the lack of need for laparotomy, which inevitably induces a systemic inflammatory response. Zigmond *et al*.^24^ reported that, using this method, the tumour incidence in the surviving mice (95%) in over 200 implantation was 100%, always culminating with a tumour development at the injection site. Major complications associated with this procedure are bowel perforation and peritoneal seeding, which are primarily dependent on the required manual dexterity. Cancer cell leak into the abdominal cavity may lead to the peritoneal seeding without the tumour formation at the primary injection site.

The biological mechanisms underlying the eNAFL irradiation of tissue have not been completely understood. Previous studies, investigating fractional laser irradiation effects on skin remodelling reported that the irradiated tissue shows local inflammation^25–27^, which can suppress tumour growth at the early tumour stages^20,28^. Similarly, our histological examinations at different time points after eNAFL treatment showed immune cell infiltration in the normal and tumour-harbouring colon tissue. Thermal injury and consequent tissue changes may induce the changes in the microenvironment of normal and tumour tissues. Tissue repairing processes after thermal damage require chemokines to recruit inflammatory cells in addition to vascular reactions. Here, we showed observed enhanced inflammatory responses in the colon tissue samples obtained from mice with and without tumour implantations. Therefore, the induced inflammation in the tumour microenvironment may have affected the observed tumour growth delay.

To understand the effects of eNAFL irradiation on the colon wall, we devised an infrared thermal imaging experiment to directly monitor the thermal effect throughout this process (Supplementary Methods). Since no thermal endoscopic instruments are available yet, we used an alternative approach, the imaging of an exposed inner colonic wall after surgical incision of the colon (Suppl. Fig. S1). After the irradiation of two sites at the luminal side of mucosal wall, mucosal focal changes were detected. Thermal images showed a rise in temperature, which then returned to the baseline temperature at the irradiation spot. The maximum temperature was reached in 2 s, by which time the spot was no longer irradiated, followed by the return of temperature to the baseline. The peak temperature change was ~20 °C. H&E staining of the tissues fixed at 1 h after the irradiation showed thermal damage, elongated nuclei, and extracted mucin content from the glandular cells. The extent of the damaged areas was 207.4 ± 17.0 μm in the mucosal layer of the colonic wall (n = 3), which was comparable to the core diameter of the irradiation fibre. However, in the orthotopic tumour model, the tumours were located in the submucosal space, and therefore, there was a minimal direct effect on the tumour cells. Further studies of combination therapeutic strategies, including those with hyperthermia^29^, are required to develop novel approaches that may increase therapeutic efficacy.

We additionally performed numerical simulations using a thick colon tissue model to understand the exposed colon tissue and the infrared thermal imaging results better (Suppl. Fig. S2). The peak temperature was shown to reach 50 °C in 2 s, and to decrease rapidly to the background body temperature. Thermal propagation to the adjacent tissue was relatively small, limiting the extent of the region in which the temperatures were over 40 °C to below approximately 300 μm, while the core of the multimode fibre encompassed 200 μm with the contact mode irradiation. The observed lesion size agreed with the results of this simulation as well. Therefore, eNAFL irradiation can be safely applied, without inducing considerable thermal damage to the adjacent tissue.

The actual thermal imaging results showed a lower peak temperature compared with that obtained by the numerical simulation. In our simulation model, simplifying assumptions were used for the boundary conditions, and the entire boundary was considered insulated and heat transfer was only the biological heat transfer. However, in experiments, the heat transfer may be more complicated: heat can be transferred to the nearby adjacent tissue, and free convection can occur through the boundary.

For the quantitative tumour evaluation, we used white light images instead of the fluorescence signals, since the fluorescence signal intensity does not correlate well with the visual grade of the tumour (Suppl. Fig. S3). We determined the mean grey values as the fluorescence signal intensities and correlated these values with the grade obtained visually using the white light image, and no significant correlation was obtained (Pearson’s correlation coefficient r = 0.045, p = 0.726, R^2^ = 0.002). This result may be explained as follows. The advanced tumours tended to bleed, and therefore, the absorption of blood may interfere with the fluorescence signal. Additionally, with the increase in the tumour size, the attenuation of fluorescence signal due to the tissue thickness can affect the resultant signal. These factors are not easily controlled, particularly in the later stages of tumour growth. Therefore, we used fluorescence signals only to confirm the presence of the cancer cells in the early stage of tumour growth and not for the quantitative tumour growth analysis. However, as the fluorescence labelling allows the tracking of cells, our model could potentially be used to investigate the eNAFL treatment effects on metastases, when our orthotopic model is confirmed to produce consistent metastases to other organs.

Due to the mucus layer of the colon, the peak temperature after irradiation may be different in other tissues. We investigated several mouse tissue samples to evaluate temperature changes, using a thermal camera (Suppl. Fig. S4), and 70 mJ eNAFL irradiation were delivered at each location. The highest temperature peak was shown to occur in the black laser alignment paper at ear, dorsal skin, external colon, and internal colonic wall site, in a decreasing order. Therefore, for the optimisation of the therapeutic dose of eNAFL, the characteristics of target tissue should be considered as well.

Laser-induced cancer therapy was shown to have different outcomes, with some studies demonstrating inhibitory effects on tumour growth^30,31^, while the others reported contrasting results^32,33^. The characteristics of tumour cells, irradiating laser types or wavelengths, or the dose of irradiation may result in different tumour responses. Therefore, eNAFL treatment requires careful evaluation using distinct tumour types with the optimised parameters.

In our study, eNAFL therapy was shown to delay tumour growth for approximately two days compared with that of the control group, before the tumours reached visual grade 3. Although this difference is small, it is significant. Bevacizumab (anti-vascular endothelial growth factor (VEGF) agent; Avastin) was shown to increase clinical survival rates^34^, and with another chemotherapeutic drug, bevacizumab prolonged the overall survival for five months, which led to obtaining FDA approval^35^. However, a growth delay of 2–3 days was observed in preclinical xenograft models treated with the anti-VEGF antibody^36^. Therefore, the tumour growth delay observed here may be translatable to the clinic, and further investigations may improve current outcomes.

In conclusion, eNAFL therapy led to a tumour growth delay when applied for the treatment of the early-stage orthotopic colon tumour. However, both the treated and control groups showed persistent tumour growth in the later stages, showing that the application of this method alone may not lead to the desirable outcomes in clinic. Furthermore, a number of parameters should be considered, and some of them have not been optimised in this study, such as laser energy, the number of irradiation spots, irradiation duration, and others. Further optimisation and potential combination with other therapeutic modalities are warranted to improve treatment efficacy. This newly developed endoscopic approach has a great potential as a minimally invasive therapeutic interventional technology that can be applied at the early stage of tumourigenesis.

## Methods

### The development of the eNAFL system

**Figure 6:**
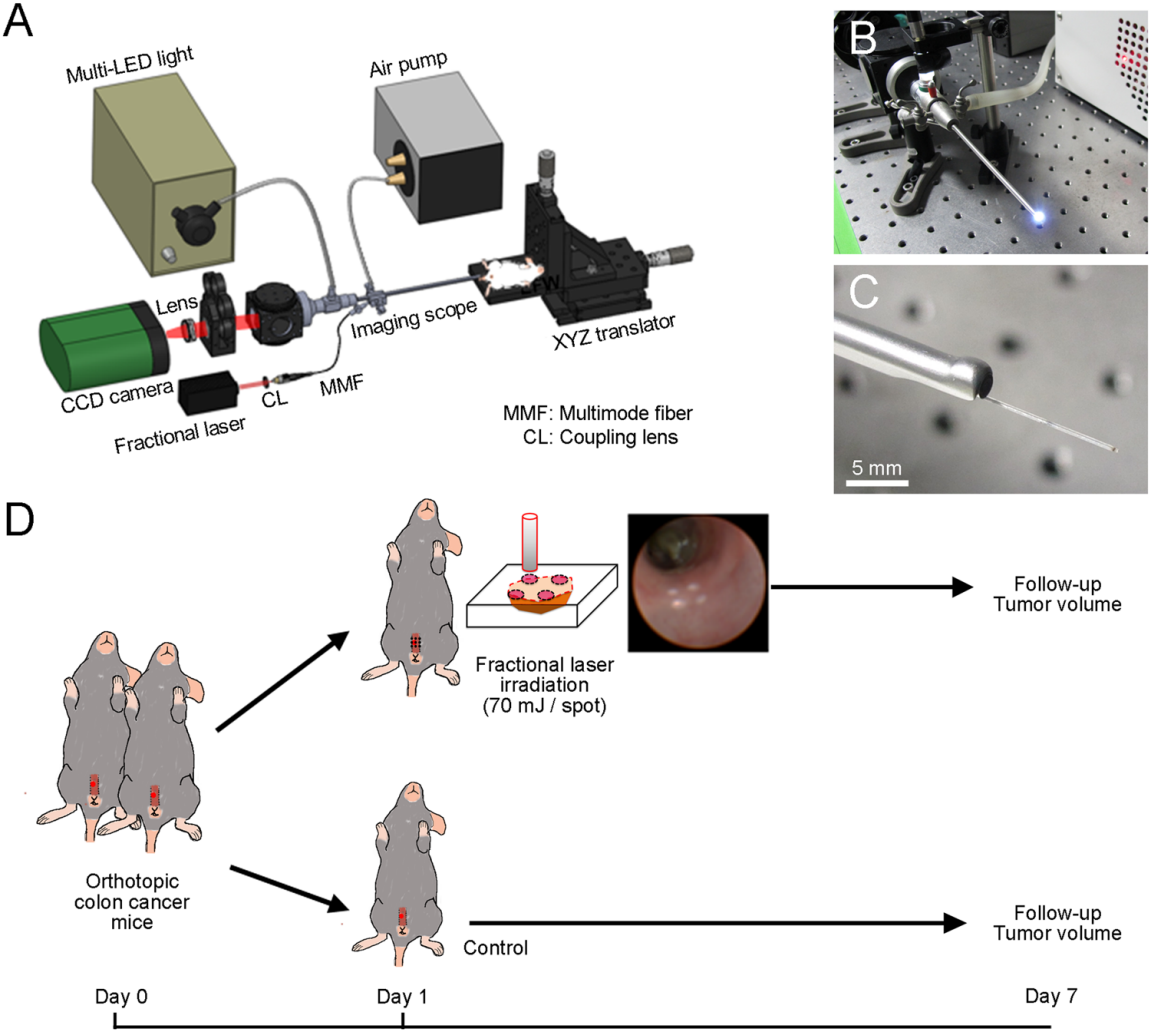
An endoscopic non-ablative fractional laser (eNAFL) system for the treatment of orthotopic mouse colon tumours. (**A**) Schematic illustration of the eNAFL system. (**B**) Images of the endoscope. (**C**) eNAFL delivering multimode fibre was inserted via the instrument channel of the endoscope. (**D**) Schematic representation of the experiment. Tumour-bearing mice were divided into the irradiated and control groups. eNAFL (70 mJ/spot) was applied in the irradiated group. Serial tumour volume was measured to estimate the therapeutic efficacy of the eNAFL irradiation for 1 week.

A thulium fibre-based fractional laser system (1927 nm, Lavieen, WONTECH, Daejeon, South Korea) was combined with a multi-channel endoscopy system (Fig. 6A and B). After disassembling the handpiece, the output of the thulium laser (continuous wave mode) was coupled to a multimode fibre (FG200LEA, Thorlabs, Newton, NJ, USA) through a fibre port coupler (PAF-SMA-11D, Thorlabs, Newton, NJ, USA). The core, cladding, and coating diameters of the multimode fibre with 0.22 NA were 200 µm, 220 µm, and 320 µm, respectively. The distal tip of the coupled multimode fibre was inserted into the instrument channel of the endoscope (Fig. 6C). The output laser power was measured using a power meter (PM100A) with an InGaAs detector (S148C, Thorlabs, Newton, NJ, USA).

### Development of the orthotopic colon tumour model

This study was approved by the Institutional Animal Care and Use Committee of the Gwangju Institute of Science and Technology (GIST-2016-32) and all experiments were performed in accordance with relevant guidelines and regulations. We attempted three different approaches in order to find an optimal orthotopic colon tumour model.

The first approach was the intraluminal seeding with laparotomy of colon cancer cells. Six-week-old female C57BL/6 mice were placed in the supine position anaesthetised with zoletil/xylazine mixture in a saline solution (60/10 mg/kg body weight). A polyethylene tube (PE10, inner diameter: 0.28 mm, outer diameter: 0.61 mm, Becton Dickinson, Sparks, MD, USA) was applied to the tip of the 30-gauge needle. After mouse laparotomy, distal colon was found with intestinal forceps. The upper portion of the colon was lightly ligated with polyamide monofilament, non-absorbable suture materials (Daflon 3-0, B. Braun Surgical, S.A. Rubi. Spain). PE10 tube tip was inserted via anus to the ligated site of the colon. Trypsin-EDTA solution (150 µL, Gibco, Invitrogen Corporation, Grand Island, NY, USA) was introduced into the lumen via PE10 tip and incubated for 10 min to irritate mucosal barrier, which was followed by washing with PBS. The distal portion of the colon was temporarily ligated using the same suture. Afterwards, 1 × 10^6^ SL4-DsRed cells in 50 µL PBS suspensions were injected via the PE10 tube into the colonic lumen. After 30-min incubation, the suture and PE10 tips were removed. Mouse peritoneum and the abdominal wall were sutured with polyglactin suture materials (Vicryl 6-0, ETHICON, Inc., Guaynabo, Puerto Rico). Ketoprofen (5 mg/kg) was subcutaneously injected for pain control.

The second approach was the intra-colonic wall injection with laparotomy of colon cancer cells. Laparotomies were performed under anaesthesia, and 1 × 10^5^ SL4-DsRed cells in 10 µL PBS were injected into the colonic wall using 28-gauge needle attached to the 10-µL Hamilton syringe (701RN, Hamilton Syringe Company, Reno, NV, USA). After the injection, mouse peritoneum and the abdominal wall were sutured as described.

The final approach was the endoscopic implantation of colon cancer cells. A home-built multi-channel mouse endoscopic system was used for the endoscopic investigation^37^. Endoscopic needle injection was described previously^24^. Under anaesthesia, the endoscopic tip was inserted into mouse anus. Eight-inch needle (30-gauge, bevelled at 45 degrees, Cadence, Inc., VA, USA) was inserted via the instrument channel of an endoscope, and 1 × 10^5^ SL4-DsRed cells in 30 µL PBS were injected.

After the orthotopic cancer cell implantation, we performed endoscopic imaging to compare tumour formation rate and the follow-up period. Successful implantation was determined by the presence of red fluorescence in the colonic wall during the follow-up endoscopic examinations, performed twice weekly. Overall tumour production success rate and the last follow-up days to tumour formation were recorded. After comparing these three orthotopic tumour implantation methods, we selected the best approach for eNAFL irradiation. Fluorescence images were obtained using a scientific colour CCD camera (QIClick, QImaging, Surrey, BC, Canada; Binning: 4, exposure time: 100 ms) and the RGB-based image was converted into a 16-bit grayscale image using the ImageJ software (NIH, Bethesda, MD, USA).

### eNAFL irradiation of the orthotopic colon tumour model

Nine mice with orthotopic tumours were randomly separated into the irradiation (n = 5) and control (n = 4) groups (Fig. 6D). All orthotopic colon tumour mice were generated using the endoscopic implantation method. All mice had Ds-Red-emitting tumour cells in the colonic wall (visual grade 0 or 1) confirmed by fluorescence endoscopy at on one day after cancer cell implantation. A total of 70 mJ energy (35 mJ/s × 2 s) were delivered to each spot via the multimode fibre tip with contact mode to the colonic wall. The irradiation group had four irradiation spots around the tumour cell accumulation (two by two manner). We used the same total irradiation energy (70 mJ/spot) as in our previous study^20^. To imitate the fractionated laser treatment, we used a simple irradiation at four spots. The control group mice did not receive any treatment. Follow-up endoscopic examinations were performed using the fluorescence and white light imaging.

### Cell culture

Murine colon cancer cells (SL4-DsRed) were cultured for the use in syngeneic xenograft tumour models. This cell line was kindly provided by the Edwin L. Steel Laboratory (Massachusetts General Hospital and Harvard Medical School, MA, USA). SL4-DsRed cells were cultured in Dulbecco’s modified Eagle’s medium/Ham’s F-12 (DMEM/F12) 1:1 medium supplemented with L-glutamine and 2.438 g/L NaHCO3, 10% foetal bovine serum (FBS), and 1% penicillin/streptomycin solution. All media and reagents were purchased from Gibco (Invitrogen Corporation, Grand Island, NY, USA).

### Numerical simulation of the temperature distribution following the eNAFL irradiation

To determine the spatiotemporal thermal effect on the colon tissue during and after NAFL irradiation, we modelled the tissue of interest as a cylindrical shape with radius of 5 mm and the thickness (h) of 500 μm. The laser beam has been modelled as a spatially distributed heat source on the surface by using a built-in Gaussian pulse function, where the beam size (diameter, 200 μm) with a standard deviation of three has been considered, which accounts for 99.7% of the total laser power (35 mW), in order to closely match the real experimental conditions. The laser irradiation lasted for 2 s on the tissue surface. The surrounding colon tissue was modelled using the extra-fine tetrahedral swept mesh method, which facilities only a single thin element through the thickness, while maintaining reasonable size in the plane. The numerical computation of the photothermal analysis of the colon model was performed using a commercially available software (Comsol Multiphysics Version 5.1, COMSOL Inc., CA, USA). Eq. (1) represents the governing equation for heat transfer through the tissue with laser irradiation:

1 $${\rm{\rho }}{C}_{p}\frac{\partial T}{\partial t}=\nabla \cdot (k\nabla T)+{Q}_{bio}+{Q}_{laser}$$

This equation represents the general form of the heat diffusion equation where T, ρ, C_p_, k, t, Q_bio_, and Q_laser_ are the temperature of tissue, density, specific heat, thermal conductivity, time, non-directional heat exchange by blood perfusion, and heat source by laser absorption into the tissue, respectively. Q_bio_ is based on the Pennes bioheat equation, presented as Eq. (2)^38^:

2 $${Q}_{bio}={\rho }_{b}\,{C}_{b}{{\rm{\omega }}}_{b}\,(T-{T}_{b})+{Q}_{m},$$

with the blood density ρ_b_, blood specific heat capacity C_b_, blood perfusion rate ω_b_, blood temperature T_b_, and metabolic heat generation Q_m_^20^. Due to the lack of colon-specific thermal properties, we used thermal properties of the skin and the blood to solve the bioheat equation. All parameters used in our tissue model are based on the information provided in our previous report^20^. The results of this simulation were displayed as a spatial and temporal distribution of temperature throughout the tissue model.

### Visual grading of the tumour growth

Tumour growth in the colonic lumen was examined using a custom-built endoscope capable of both white light and fluorescence imaging. Endoscopic scoring of tumour development was done according to the methods described in the previous reports^39,40^. Briefly, tumour size was graded as follows: grade 1, very small but detectable tumour; grade 2, tumour covering up to one-eighth of the colonic circumference; grade 3, tumour covering up to a quarter of the colonic circumference; grade 4, tumour covering up to one half of the colonic circumference; and grade 5, tumour covering more than a half of the colonic circumference. Additionally, we added grade 0 to this scale, representing tumours undetectable by using white light, but with the detectable cancer cell fluorescence signal. Tumour grade was assessed by the blinded personnel experienced in preclinical endoscopy. Statistical analyses were performed to evaluate the difference in the tumour grade between the eNAFL-irradiated group and the control group, using Mann-Whitney U test. Statistical analyses were performed using GraphPad Prism 6 (GraphPad Software Inc., La Jolla, CA, USA).

### Histological analysis of the tissue changes following the eNAFL treatment

To evaluate the tissue responses to the eNAFL irradiation, histological analyses of both control and tumour-bearing mice were performed. Control and tumour-bearing mouse colons were extracted before, 6 h after, and 48 h after eNAFL irradiation. Tissue changes and immune cell infiltration rates were evaluated using the H&E staining.

### Data availability statement

The datasets generated and/or analysed during the current study are available from the corresponding author upon reasonable request.

## Electronic supplementary material

Supplementary Video S1

Supplementary information
