# Supplementary material for: Endoscopic non-ablative fractional laser therapy in an orthotopic colon tumour model
Source: Sci Rep. 2018 Jan 26;8:1673. doi: 10.1038/s41598-018-19792-2 (PMC5785993; doi:10.1038/s41598-018-19792-2)
Supplement: Supplementary file 2 — Supplementary information [file 41598_2018_19792_MOESM2_ESM.doc]

Supplementary information

Endoscopic non-ablative fractional laser therapy in an orthotopic colon tumour model

Su Woong Yoo^1,2^, Gyungseok Oh^3^, Abdul M. Safi^2^, Soonjoo Hwang^2^, Young-Seok Seo^4^, Kyung-Hwa Lee^5^, Young L. Kim^6^, and Euiheon Chung^2,3*^

^1^Department of Nuclear Medicine, Chonnam National University Hwasun Hospital, Jeollanam-do, Republic of Korea

^2^Department of Biomedical Science and Engineering, Institute of Integrated Technology (IIT), Gwangju Institute of Science and Technology (GIST), Gwangju, Republic of Korea

^3^School of Mechanical Engineering, Gwangju Institute of Science and Technology (GIST), Gwangju, Republic of Korea

^4^R & D center, WONTECH Co., Ltd., Daejeon, Republic of Korea

^5^Department of Pathology, Chonnam National University Hwasun Hospital and Medical School, Jeollanam-do, Republic of Korea

^6^Weldon School of Biomedical Engineering, Purdue University, IN, USA

^*^Correspondence and requests for materials should be addressed to E. C. (ogong50@gist.ac.kr).

**Supplementary Methods**

**Thermal imaging to monitor temperature change with eNAFL irradiation.** To confirm the validity of our numerical simulation, we devised an experiment using eNAFL irradiation on exposed colon tissue with real-time thermography after open-belly surgery (Supplementary Figure S1). The thermal infrared imaging experiments were performed in a room at a constant temperature of typically 23 °C. The thermal camera (FLIR A325sc, FLIR system, USA) contains 320 × 240 pixels with a spectral response range of 7.5 – 13 μm. The noise equivalent temperature difference (NETD) is 50 mK, and temperature measurement accuracy is ± 2 °C. The experimental procedure consists the following steps. First, the steady-state temperature distribution in the specimen area was recorded using the thermal camera for 5 seconds. Next, fractional laser light from the multimode fibre was irradiated followed by measurement of temperature transients on the tissue surface with the same parameters as the colonic eNAFL treatment study (35 mJ/sec × 2 sec). The total time for thermal imaging was 20 seconds with 15 frames per seconds. Finally, quantitative analysis of irradiated tissue was assessed using FLIR ResearchIR Max software (FLIR System, USA).

**Supplementary Videos**

**Supplementary Video S1. Endoscopic implantation of colon cancer cells.**

**Supplementary Figures**


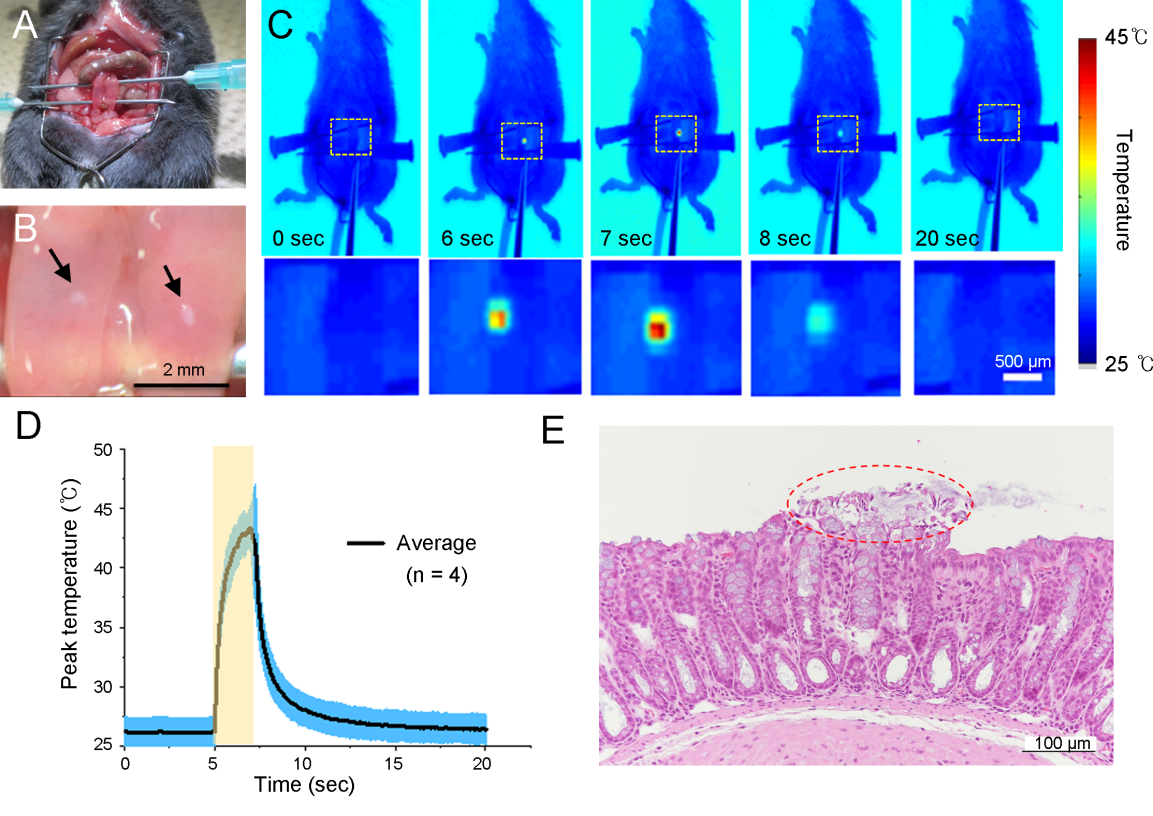


**Supplementary Figure S1. Real-time thermal imaging of the exposed colon with eNAFL irradiation.** (A) Mouse colon was exposed for a surgical procedure. (B) Affected colonic wall just after endoscopic non-ablative fractional laser (eNAFL) irradiation., (C) Serial thermal images of mouse colon during observation time. Lower row showed magnified images of the irradiated area (yellow-dotted rectangular area). (D) Average temperature changes before (0 - 5 sec), during (5 - 7 sec) and after (7 - 20 sec) irradiation. Blue lines showed standard deviations (n = 4 spots of irradiation). (E) Hematoxylin & Eosin (H & E) staining of the tissue with eNAFL irradiation. The thermally damaged lesion was marked with the red dotted circle.


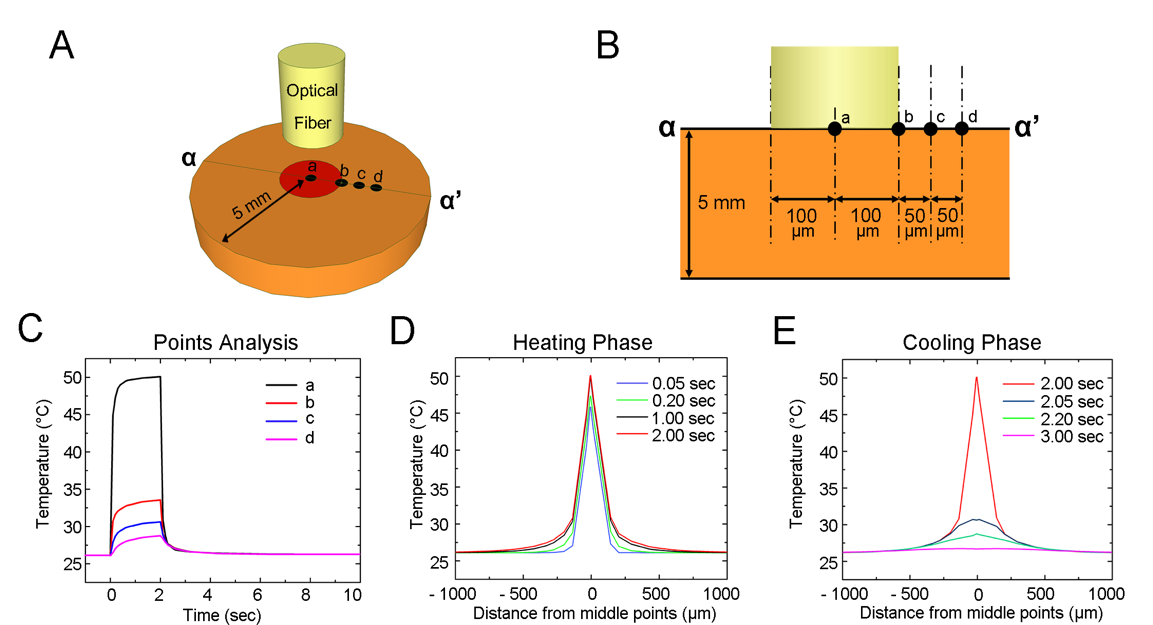


**Supplementary Figure S2. Numerical simulation of endoscopic non-ablative fractional laser (eNAFL) irradiation to the exposed colon.** (A) 3D image of the tissue model for numerical simulation. (B) Cross-cut image of the 3D tissue model with plane α–α’ in Fig. 8A. Note the thickness of the tissue is 5 mm to represent a semi-infinite medium. (C) Temporal analysis of four representative spots. (D, E) Spatial distribution of the temperature during heating (D) and cooling (E) in the tissue model.


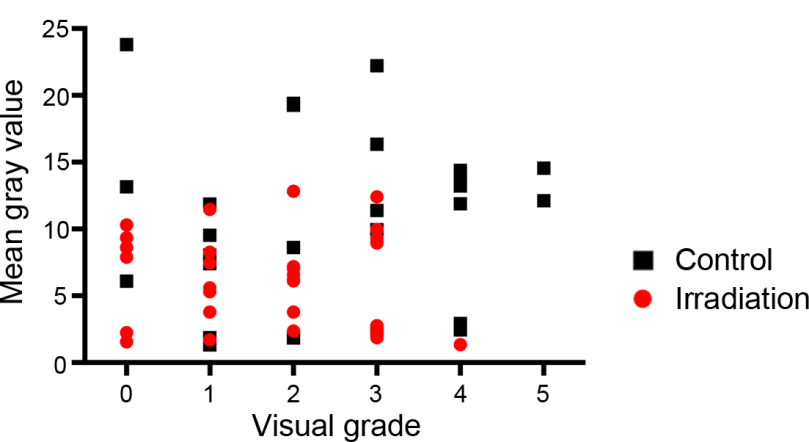


**Supplementary Figure S3. Correlation between visual grading and fluorescence signal intensities in orthotopic colon tumour model.** There was no significant correlation between two parameters in both control (black rectangle) and irradiation (red dot) groups. (Pearson’s correlation coefficient r = 0.045, p = 0.726, R^2^ = 0.002)


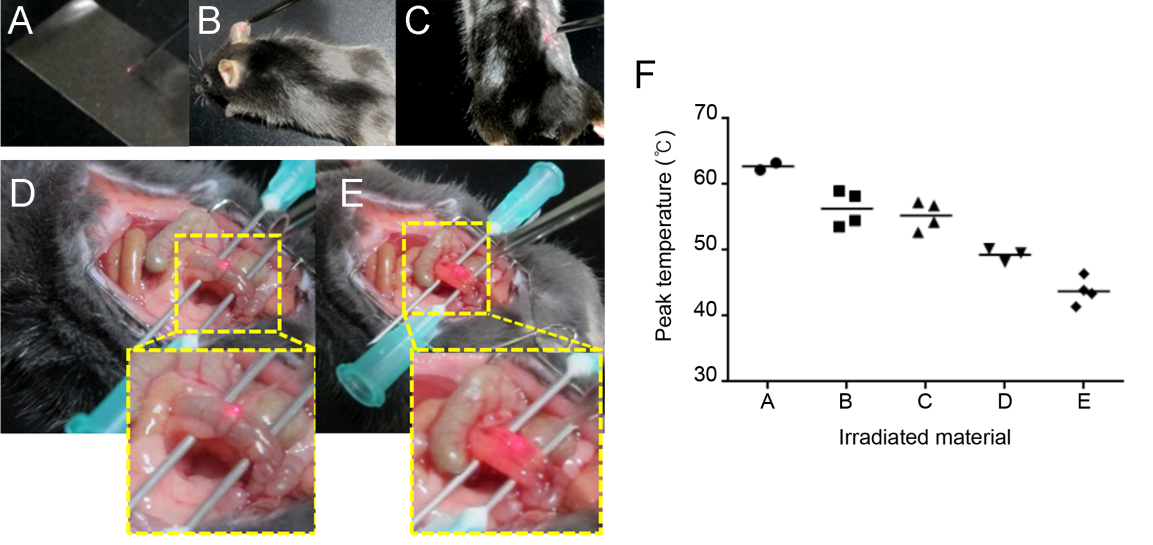


**Supplementary Figure S4. Peak temperature in various materials.** Same energies of the endoscopic non-ablative fractional laser (eNAFL) was irradiated to (A) laser alignment paper (Zap-It, Kentek Corp., Pittsfield, NH, USA) (B) ear, (C) dorsal skin, (D) external (visceral side) colonic wall and (E) internal colonic wall of C57BL/6 mouse. (F) Peak temperature in each irradiated material.
